# Effect of Ketamine on Sleep in Treatment-Resistant Depression: A Systematic Review

**DOI:** 10.3390/ph16040568

**Published:** 2023-04-10

**Authors:** Aleksander Kwaśny, Adam Włodarczyk, Damian Ogonowski, Wiesław Jerzy Cubała

**Affiliations:** Department of Psychiatry, Faculty of Medicine, Medical University of Gdansk, 80-214 Gdańsk, Poland

**Keywords:** ketamine, esketamine, arketamine, ketamine metabolites, insomnia, hypersomnia, circadian rhythm, depression, major depressive disorder

## Abstract

Background: Depression is a debilitating disease with a high socioeconomic burden. Regular antidepressants usually require several weeks to ameliorate symptoms; however, numerous patients do not achieve remission. What is more, sleep disturbances are one of the most common residual symptoms. Ketamine is a novel antidepressant with rapid onset of action with a proven antisuicidal effect. Little is known about its impact on sleep–wake and circadian rhythm alterations. The aim of this systematic review is to research the impact ketamine has on sleep disturbances in depression. Methods: PubMed, Web of Science, and APA PsycINFO were searched for relevant studies on ketamine’s impact on sleep disturbances in depression. Preferred Reporting Items for Systematic Reviews and Meta-Analyses PRISMA2020 methodology was applied. The systematic review protocol was registered in the PROSPERO Registry (CRD42023387897). Results: Five studies were included in this review. Two studies reported significant improvement in sleep measured by MADRS (Montgomery–Åsberg Depression Rating Scale) and QIDS-SR16 (Quick Inventory of Depressive Symptomatology Self-Report (16-item)) scales after intravenous ketamine and intranasal esketamine administration. One case report showed mitigation of symptoms in PSQI (Pittsburgh Sleep Quality Index) and ISI (Insomnia Severity Index) during 3-month treatment with esketamine. In two studies, sleep was objectively measured by nocturnal EEG (electroencephalography) and showed a decrease in nocturnal wakefulness accompanied by an increase in slow wave (SWS) and rapid eye movement (REM) sleep. Conclusion: Ketamine reduces the severity of sleep insomnia in depression. Robust data are lacking. More research is needed.

## 1. Introduction

Major depression carries a great burden and occupies a top-ranking place among diseases with a leading cause of disability worldwide according to World Health Organization (WHO) registries [1,2]. The incidence of this mental disorder is estimated to be around 350 million people worldwide [3,4]. People affected by the disease suffer a great personal cost, as does the community.

Following the STAR*D trial, only about a third of patients experience remission of symptoms with first-line antidepressants, and the cumulative rates were about two-thirds for four consecutive lines of treatment [5,6]. These results show that approximately 30% of patients are treatment-resistant and are left without satisfactory outcomes when treated with regular antidepressants [7,8,9].

Due to the heterogeneous nature of the disease, a myriad of treatment-resistant depression (TRD) definitions is in use. TRD is commonly defined as a failure of at least two antidepressant therapies (ADT) given at an adequate dose for an adequate time during a current major depressive disorder (MDD) [10,11]. Although two failed trials is the most common number, other definitions raise the threshold even up to five trial failures. This might include antidepressants from the same class yet may also require a switch to a different antidepressant class, pharmacological augmentation, or non-pharmacological intervention [7,12]. Furthermore, the adequacy duration is highly variable with a timeframe varying between 4 and 12 weeks. In addition, even up to 70% of studies fail to report the definition of the adequacy of the trial duration [12,13]. Despite not being well-presented in diagnostic criteria, an adherence level of 80% enables the trial to be considered valid. This poses a clinical and research challenge and non-adherence may be contributory to TRD [10,11]. Not all depressive episodes are resistant to regular therapeutic options, but four distinctive predictors are known: comorbid anxiety disorder, suicidality, the severity of the depressive episode, and the number of previous episodes. MDD at an early age, psychotic depression, long duration of depressive episodes, a number of previous hospitalizations, delayed treatment initiation, and sociodemographic factors (e.g., gender or educational level) are also linked to treatment resistance. Other non-psychiatric risk factors include hypothyroidism or vitamin deficiencies [7,14,15].

Ketamine is a rapid-acting antidepressant. It is a racemic mixture of (*R*)-ketamine (or arketamine) and (*S*)-ketamine (or esketamine) [16]. Both isoforms perform *N*-methyl-D-aspartate (NMDA) receptor antagonism, with the (*S*)-enantiomer having a greater affinity for the receptor than the (*R*)-enantiomer [16]. In 1964, it was first administered in humans to induce anaesthesia as an alternative to phencyclidine [17,18,19]. It was later approved as a rapid-acting general anaesthetic in 1970 with possible intravenous or intramuscular administration routes [20]. In 2000, Berman and colleagues at Yale University observed robust mitigation of depressive symptoms in patients with major depressive disorder (MDD) treated with intravenous ketamine hydrochloride in a dose of 0.5 mg/kg [21]. Subsequently, in 2019 the Food and Drug Administration (FDA) approved esketamine for treatment-resistant depression (TRD) [22]. Previous research suggests that the antidepressant effect is immediate and emerges within approximately 2 h [23]. In addition, there is evidence that a single infusion of ketamine reduces suicidal ideation in the short term [24]. Moreover, it possesses antianhedonic and possibly procognitive properties [25,26].

Ketamine enantiomers block NMDA receptors on GABA inhibitory interneurons. Consequently, de-inhibition of pyramidal neurons increases the output of excitatory glutamate neurotransmitter, which in turn activates AMPA receptors leading to an increase in the brain-derived neurotrophic factor (BDNF) [27]. BDNF is a neuropeptide with a high expression in the central nervous system that plays an essential role in neuroplasticity [28]. Ketamine-induced changes in BDNF levels correlate with both sleep slow waves (SWS) and mood fluctuations, such as poor quality of sleep in subjects with treatment-resistant depression (TRD) [29]. Furthermore, circadian locomotor output cycles kaput (*CLOCK*) is a gene encoding a basic helix-loop-helix-Per-Arnt-Sim transcription factor that is known to affect both the persistence and period of circadian rhythms [30]. In humans, some polymorphism in *CLOCK* may be related to the recurrence of major depressive episodes in patients with bipolar disorder, increased insomnia, and diurnal preference [31,32,33]. There is evidence of ketamine’s effects on *CLOCK* genes to influence circadian timing and on BDNF and SWS to affect sleep quality [34].

Circadian rhythms and sleep–wake alterations, as defined by Research Domain Criteria, are one of the diagnostic criteria for MDD [35]. Sleep–wake alterations are prevalent not only in the acute phase of the disease but also as residual symptoms and are highly related to disease relapse in the future [36,37,38]. Disrupted sleep is a common complaint, and adequate sleep is imperative in achieving quality remission. Yet the onset of clinical effects of regular antidepressants is delayed, and some of them contribute to the disruption of sleep [39]. Despite considerable progress in ketamine research, there is still limited data on ketamine’s impact on sleep disturbances in treatment-resistant depression [34]. Therefore, this systematic review aims to answer the following question:

**Does ketamine have an impact on sleep disturbances in treatment-resistant depression?**

## 2. Results

### 2.1. Study Selection

**Figure 1:**
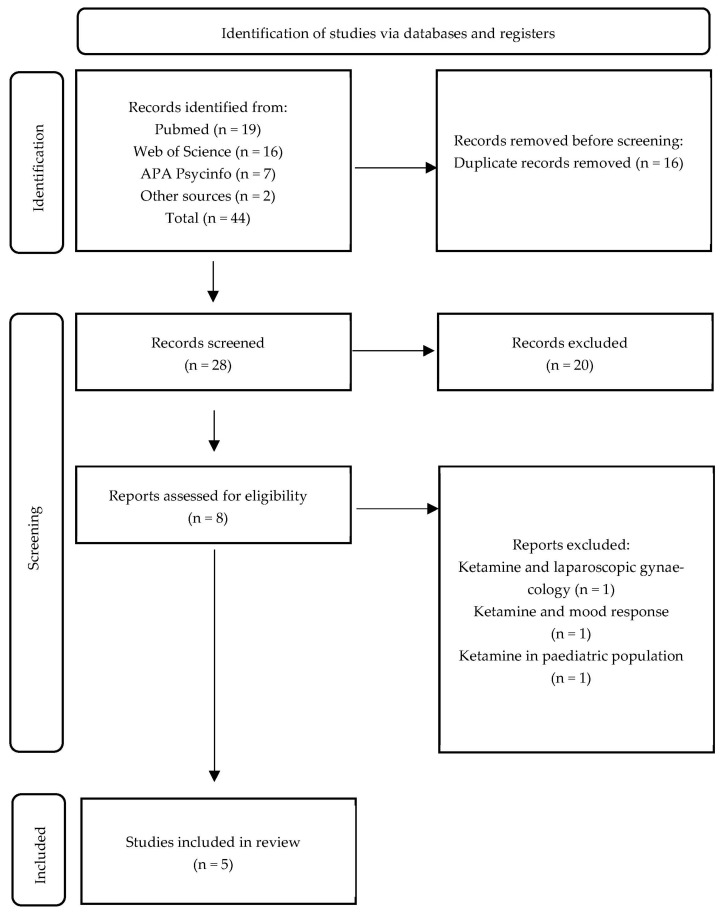
Flow chart representing the search strategy and the process of including studies for analysis.

A total of 44 citations were identified. A detailed screening process is presented below in the form of a PRISMA flow chart (Figure 1). Five studies were included in the study: one randomised controlled trial (RCT), two post hoc analyses of RCTs, one post hoc analysis of a naturalistic study, and one case report. No RCTs with sleep outcomes as the main target were found. All studies included are listed in Table 1.

### 2.2. Risk of Bias in the Studies

**Figure 2:**
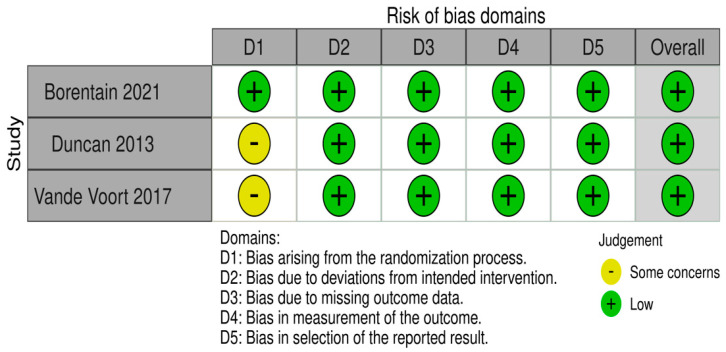
Risk of bias for randomised studies with the RoB 2 tool [29,41,42].

Studies with randomisation were assessed according to the RoB 2 tool (a revised tool for assessing the Risk of Bias in randomised trials). Two studies did not fully describe the randomisation process, yet the differences in both groups were of no statistical significance and the groups were merged for statistical analysis; therefore, all three studies were ranked as “low risk of bias” [41,42,43]. One non-randomised naturalistic study was evaluated using the Newcastle–Ottawa Scale and was assessed as a “good quality” study [40]. Outcomes for all studies are presented in Figure 2 and Table 2.

### 2.3. Study Characteristics

#### 2.3.1. Self-Reported and Clinician-Rated Sleep Patterns

The post hoc analysis by Rodrigues and colleagues [40] describes a naturalistic group of patients referred to the Canadian Rapid Treatment Center of Excellence who received intravenous ketamine in a dose of 0.5 mg/kg in two initial infusions. The dose was maintained in the following two infusions unless there was no sufficient clinical response (≤20% reduction in total score in QIDS-SR16 (Quick Inventory of Depressive Symptomatology Self-Report [16-item]). In that case, the ketamine dose was increased to 0.75 mg/kg in the remaining one or two infusions. Treatment with ketamine was significantly associated with a reduction in three sleep items in QIDS-SR16 (insomnia, night-time restlessness, and early-morning waking). In hypersomnia, the positive effect of infusions was observed; however, it was of no statistical significance. Moreover, a reduction in any of the sleep items (including hypersomnia) and total sleep score was considered a partial mediator of the improvement in depression severity with either a small (single sleep item) or medium (total sleep score) effect size. Improvement in sleep had an additionally small but significant effect on the reduction of suicidal ideation. Change in total sleep score was determined to be the only predictor of reaching response or remitter status among the ones tested, with greater improvement in sleep being associated with an increased likelihood of achieving response or remission.

A retrospective analysis [41] investigated the effect of esketamine nasal spray in outpatients with treatment-resistant depression in two similarly designed, 4-week, double-blind, randomised, active-controlled clinical trials. In the first study, patients received a fixed dose (either 56 mg or 84 mg), whereas in the second, the dose was flexible depending on the efficacy and tolerability. The assessment was carried out by blinded, remote raters on the MADRS scale. Sleep complaints were measured using MADRS (Montgomery–Åsberg Depression Rating Scale) item 4. At the entrance, most patients reported sleep disturbances. Among them, almost two-thirds described their sleep as either moderately or severely disturbed. Improvement in sleep was observed within the first 24 h after the first ketamine infusion, in both esketamine and placebo groups. Nevertheless, amelioration of sleep difficulties in the esketamine group was more distinctive on day 8 and at all subsequent evaluations. Reduction of two or more points in MADRS item 4 (described by authors as a clinically meaningful response) was more common in the active treatment group from day 8 until the end of the double-blind treatment period. If only patients with ≤4 MADRS item 4 score were considered, improvement (score 3 or less) was more often reported in the esketamine group (42.5%) than placebo (28.7%) at day 1 post ketamine infusion, whereas at day 28 there was no difference as over two-thirds in both groups improved.

Another study is a case report of a 55-year-old woman with treatment-resistant depression and unsatisfactory response, despite having tried fluoxetine, duloxetine, escitalopram, and bupropion previously. She complained about insomnia, loss of energy and interest, and decreased concentration, motivation, libido, and self-esteem. In the Beck Depression Inventory (BDI), her score was 38 despite two concomitant antidepressants. Treatment with intranasal esketamine was initiated and continued for 3 months. Apart from a decrease in BDI from 38 to 9, her scores on Pittsburgh Sleep Quality Index (PSQI) reduced from 13 to 8 and on Insomnia Severity Index (ISI) from 11 to 7 over the course of the therapy [43].

#### 2.3.2. Electroencephalographic Sleep Patterns

In another post hoc analysis with 34 depressed patients (23 MDD, 11 bipolar depression) and 22 healthy controls, nighttime EEG was carried out the day before and the day after a single intravenous ketamine infusion (0.5 mg/kg over 40 min). Patients with MDD were medication-free and patients with bipolar disorder (BPD) had either lithium or valproate at therapeutic levels. Furthermore, 11 patients were randomised to receive add-on riluzole treatment. Since the overall results were comparable, the entire group (ketamine only vs. ketamine + riluzole) was considered as one entity. Suicidal ideation was measured using the Hamilton Depression Rating Scale, where the antisuicidal response was defined as a score of 0. Nocturnal wakefulness was defined as minutes awake in the time frame between midnight and 4:59 AM. Suicide responders spent significantly fewer minutes awake at night than non-responders. Moreover, results suggested potential differences in the average number of minutes awake at night between healthy controls and participants with an antisuicidal response [42].

Similarly, as in the previous study, 30 treatment-resistant MDD patients without antidepressant therapy for at least 2 weeks received a single intravenous ketamine infusion of 0.5 mg/kg over 40 min. Patients were then randomised to receive 4–6 h post-infusion either riluzole (*n* = 19) or placebo (*n* = 11). BDNF was collected before and 230 min after infusion. Nocturnal electroencephalography was conducted the night before and two nights after the infusion. Since there were no statistical differences for either group (ketamine/placebo vs. ketamine/riluzole), the entire sample was analysed as a single group with a single infusion of intravenous ketamine. In comparison to the baseline night, a significant improvement could be noticed in total sleep time, slow wave sleep, and REM sleep. Consolidation of sleep was observed after ketamine infusion defined as a reduction in S1, S2, REM latency, and waking time. Total slow wave activity amount increased, especially in the first NREM sleep cycle. The EEG frequencies associated with slow waves increased significantly. The slow wave amplitudes were redistributed towards high amplitudes, while the overall number of slow waves was not influenced by ketamine administration. Nonetheless, only the responder group (*n* = 13) showed a significant correlation between slow wave activity and log BDNF, which could not be seen in the non-responder group [29].

## 3. Discussion

This systematic review supports the evidence in favour of ketamine use in treatment-resistant depression with sleep disturbances as part of major depressive disorder and bipolar depression. The relationship between sleep disruption and mood disorders is bidirectional, which means sleep impairment may precede mood symptoms or depressed mood can bring about sleep disruption [27]. Moreover, ketamine’s effect on sleep should be taken into consideration from both an antidepressive and antisuicidal perspective.

As shown by Rodrigues and colleagues, change in sleep patterns plays a small to moderate role in reducing the severity of the episode in subjects receiving intravenous ketamine infusions. The amelioration of depression symptoms may be due to isolated improvement in insomnia symptoms or the changes may function as a mediator of overall depression severity reduction. Moreover, improvement in overall sleep score was the only predictor of achieving responder/remitter status, with an increased likelihood of achieving the status with greater improvement in the sleep pattern [43]. In MDD patients with rapid antidepressant response, the improvement might be linked to sleep changes such as decreased waking and an increase in the following: total sleep (TS), slow wave sleep, slow wave activity (SWA), and REM sleep [29]. Synaptic strength and cortical synchronisation are functionally related to sleep slow wave biomarkers. The correlation of SWS biomarkers and BDNF in the ketamine responders group suggests that ketamine-induced BDNF changes stimulate the synaptic strengthening [29]. Furthermore, normalising the deficient early night production of SWS plays a key role in the rapid reduction of depressive symptoms [44].

Suicidal attempts are associated, among others, with self-reported sleep difficulties [45]. Nocturnal wakefulness, especially in the early morning hours, was found to be associated with suicidal thoughts the next day [46]. Reduction of nighttime wakefulness in suicide responders emphasizes the importance of circadian rhythms in antisuicidal treatment and prevention [41]. Interestingly, in Finland, the suicide mortality rate is the highest during the period with the longest duration of the day, which can further support the evidence for circadian misalignment in suicidal behaviour [47]. Ketamine’s rapid antisuicidal effect is independent of its antidepressant outcome [48]. Glutamate is crucial in the regulation of the central clock with the light–dark cycle. Ketamine changes glutamate levels through NMDA blockade and might alter circadian activity patterns [44], which may to some extent explain antisuicidal properties. Therefore, the chronotherapeutic properties of ketamine might play a key role in explaining its antisuicidal features. This would be in line with existing evidence that chronotherapy may be linked to the mitigation of suicidal ideation (SI); for instance, rapid reductions in SI after sleep deprivation in conjunction with light therapy and the use of lithium or after sleep deprivation with cognitive-behavioural therapy [49,50].

Despite the therapeutic value of ketamine administration in a TRD population with sleep disturbances, the availability of the therapy may be limited to the clinical setting due to the ketamine’s reinforcing properties (i.e., dissociative and euphoria effects) and abuse or addiction potential [51].

While interpreting this review, one should consider the following limitations. Firstly, there were several outcome measures with each of them possibly explaining to some extent the overall effects. Regardless of their significance, it ultimately impedes the comparability among chosen studies. Secondly, some studies differed regarding concomitant antidepressant therapy. Some drugs may disrupt sleep (e.g., fluoxetine, venlafaxine), while others with sedative properties (e.g., mirtazapine, doxepin) may improve it. Antidepressants might impact sleep in a variety of ways, e.g., induce or worsen primary sleep disorders such as nightmares, sleep bruxism, restless leg syndrome, REM sleep behaviour disorder, or sleep apnoea (secondary to antidepressant-induced weight gain) [52]. Thirdly, included studies used objective and patient-reported outcomes, which on the one hand is the strength of this review, but on the other limits the plausible comparison considering the scarcity of available data. Fourthly, hypnotics and benzodiazepine use might have been confounding factors, yet it was not clear if they were allowed in some of the studies. Fifthly, the variability of sample groups must be borne in mind, as MDD only versus MDD + BPD groups might produce disparate outcomes. Sixthly, sample sizes vary distinctively, which affects the possible comparability. Seventhly, the population comprised treatment-resistant subjects; thus, the effects on sleep may not be generalisable to non-treatment-resistant patients. Eighthly, sleep disturbances per se were not the primary outcome of the included studies. In some cases, they were analysed in post hoc analyses; in others, they were related to the primary target, for instance, suicidal ideation. Finally, it must be clearly stated that this systematic review does not primarily focus on ketamine’s antisuicidal and antidepressant properties, despite their link to sleep–wake and circadian rhythm alterations.

Ketamine seems to resolve insomnia complaints, yet the existing data, however scarce, do not support its effect on hypersomnia. Still, it is impossible to determine whether sleep changes follow the global response pattern or occur independently. It may be hypothesised that residual sleep symptoms may indicate poor outcomes, which would be in line with residual cognitive deficits [26,53,54]. Another hypothesis could be that sleep changes may be indicative of ketamine and its enantiomers’ treatment prognosis in the short and long term, as some evidence in favour of electrophysiology findings support the concept. Contrary to traditionally acting antidepressants per ketamine mode of action, sleep parameters may be predictive of relapses and indicative of high-quality remission. More research and follow-up are needed.

The results shall be treated with caution as they apply to intravenous and nasal spray formulations with recent advances in clinical trials with other formulations, in particular extended-release tablets, as well as practitioners’ guidance on oral solution use. Future research shall include the factor of formulation per outcome assessed. 

No systematic prospective, interventional studies examining the impact of ketamine on sleep in TRD were carried out. The majority of data stems from exploratory post hoc analyses. Current literature data report with a variability of the outcome measures and inhomogeneity of the group studied that may produce confounding conclusions. The above-stated gaps in the literature are the key concern when selecting the data synthesis methodology. In such case, meta-analysis may produce inconclusive or confounding results and systematic review, with all its limitations, is less prone to the biases impacting conclusions. Given the scarce but promising results of ketamine’s impact on sleep and the interplay between sleep disturbances and circadian rhythms in depression, it seems worth exploring broader sleep symptomatology after ketamine administration using sleep scales such as PSQI, Epworth Sleepiness Scale, Morningness–Eveningness Questionnaire, and/or polysomnography.

## 4. Materials and Methods

This systematic review was written according to the Preferred Reporting Items for Systematic Reviews and Meta-Analyses (PRISMA) statement [55]. For PRISMA checklist and raw search results please refer to Supplementary Materials. The systematic review protocol was registered in the PROSPERO Registry (CRD42023387897). Details of the protocol for this systematic review can be accessed at https://www.crd.york.ac.uk/prospero/display_record.php?ID=CRD42023387897 (accessed on 4 April 2023). 

### 4.1. Information Sources, Search Strategy and Selection Process

On 5th January 2023, we searched PubMed, Web of Science, and APA PsycINFO electronic databases. Searches were re-run prior to submission. The following entries were used in various combinations: “insomnia”, “hypersomnia”, “ketamine”, “esketamine”, “arketamine”, “depression”, “major depressive disorder”, and “bipolar depression”. We did not apply any restriction to the date of publication.

The inclusion criteria were as follows:

1. Primary research articles;
2. Patients had either major depressive disorder or bipolar depression according to DSM or ICD criteria (without restrictions on editions);
3. Participants were exposed to ketamine or its enantiomers;
4. Pre- and posttreatment sleep outcome was available;
5. Patients were over 18 years of age.

Excluded articles did not focus on the topic of this review and investigated ketamine in laparoscopic gynaecology (1), ketamine and mood response (1), and ketamine in the paediatric population (1).

### 4.2. Data Collection Process

Two reviewers (A.K., A.W.) conducted the search process, screened abstracts and titles, and subsequently read eligible full-text articles. All disparities were discussed with project co-supervisor (W.J.C.) until an agreement was reached.

The following data were extracted: authors, year of publication, study design, sample and control size, duration of the study, characteristic of the research and control group (sex, diagnosis, treatment), dose and formulation of ketamine treatment, and outcomes (mean changes in sleep domain assessed per clinical scales or objective methods such as electroencephalography (EEG)).

### 4.3. Study Risk of Bias Assessment

The risk of bias for randomised trials was evaluated using a method recommended by Cochrane System Reviewer Manual 5.1 (a revised tool to assess the risk of bias in randomised trials—RoB 2), including sequence generation, allocation concealment, blinding, missing outcome data, selective reporting, and other bias. The risk of bias may be assessed as “low”, “some concerns”, or “high” [56,57]. To assess the risk of bias in non-randomised studies with intervention, we used the Newcastle–Ottawa Scale [58]. This scale awards a maximum of nine points for each of the following items: selection (four stars), comparability (two stars), and outcomes (three stars). Studies with a score equal to or above seven points are considered “good quality” studies. At least two independent reviewers assessed the risk of bias for each study (A.K., A.W). Conflicting information was discussed to reach an agreement with the aid of the project co-supervisor (W.J.C.). The Robvis tool was used to illustrate the results from randomised trials [59].

## 5. Conclusions

Ketamine contributes to the reduction of insomnia symptoms with potentially no effect on hypersomnia. Sleep changes mediate antidepressive and antisuicidal response. Improvement in EEG measures (SWS, SWA, TS time, and nocturnal wakefulness) was observed. Data on sleep patterns in patients exposed to ketamine are scarce with predominantly sleep data as a secondary outcome. This study should be considered as a pilot study to elucidate sleep problems in TRD patients. More clinical and experimental data are needed with sleep as a primary outcome measure.

## Figures and Tables

**Table 1:** Studies on ketamine’s impact on sleep patterns.

Author	Study Design	Number of Subjects	Population	Intervention and Route	Sleep Outcome Measures	Outcome
Rodrigues et al. 2022 [40]	Post hoc analysis Mean	323 (175 female) 275 MDD 48 BPD	Treatment-resistant depression as part of major depressive disorder or bipolar depression Treatment resistance defined as failure of two or more adequate trials of different antidepressant classes	Four infusions of intravenous (IV) ketamine dosed at 0.5 mg/kg delivered over 40–45 min; dose increased to 0.75 mg/kg if no clinical response observed	QIDS-SR16	Significant main effect of infusion on improvement in: insomnia, *p* < 0.001, effect size not stated; night-time restlessness, *p* = 0.007, effect size not stated; early morning waking, *p* = 0.04, effect size not stated; Non-significant effect of infusion on hypersomnia, *p* = 0.10 effect size not stated
Borentain et al. 2021 [41]	Post hoc analysis Mean ± SD Odds Ratio (95% CI)	565 (379 female)	Treatment-resistant depression as part of MDD Treatment resistance defined as non-response to an adequate (dose and duration) course of at least two oral antidepressants during the current depressive episode	ADT + esketamine nasal spray in fixed or flexible dose; ADT + placebo	MADRSitem no. 4	Significant improvement per MADRS item 4 in sleep disturbances in ketamine + ADT group compared to ADT standalone at every timepoint: on day 8 (*p* = 0.001, effect size not stated) and at all subsequent evaluation timepoints through day 28 (*p* = 0.020, effect size not stated)
Vande Voort et al. 2017 [42]	Post hoc analysis Mean ± SEM	34 (14 female) 23 MDD 11 BPD 22 healthy controls	Treatment-resistant depression in MDD or BPD Subjects must have failed to respond to an adequate dose and duration of at least one antidepressant (SSRI, bupropion, or venlafaxine) during a depressive episode	Intravenous ketamine hydrochloride (0.5 mg/kg over 40 min) At least two weeks without psychotropic therapy (5 weeks for fluoxetine) prior to ketamine administration. Patients with BPD were medicated with either lithium or valproate. A subset of eleven patients received a single dose of riluzole. Analyses were conducted for both riluzole + ketamine and ketamine only. Since differences were not significant, analyses were conducted for the entire sample.	Nighttime electroencephalography (EEG) the night before and the night after a single ketamine infusion	Significant reduction of nocturnal wakefulness in suicide responder group (*p* = 0.04, d = 0.96) A trend between suicide responders to healthy controls (*p* = 0.08; d = 0.56), suggesting potential difference in mean minutes awake by hour of night
Duncan et al. 2013 [29]	Randomised controlled trial Mean ± SEM	30 (10 female)	Treatment-resistant MDD TRD defined as a current or history of non-response to two adequate antidepressant trials	Ketamine + riluzole 100 mg/d; Ketamine + placebo Single IV infusion of 0.5 mg/kg ketamine hydrochloride over the course of 40 min At least two weeks without psychotropic therapy (5 weeks for fluoxetine) prior to ketamine administration. Groups were subsequently merged for statistical analysis, since riluzole had no statistical significance when it came to sleep outcome.	Nighttime EEG the night before and two nights after the infusion	Increase in total sleep time, slow wave sleep, and REM sleep (*p* < 0.05) and slow wave activity (*p* < 0.01) the night after single ketamine infusion. Reduction in S1, S2, REM latency and waking time the day after infusion (*p* < 0.05)
Stultz et al. 2020 [43]	Case report	1	Treatment-resistant depression Unsatisfactory response, despite taking fluoxetine, duloxetine, escitalopram, and bupropion in the past	Esketamine nasal spray up to 84 mg	ISI PSQI	PSQI score decreased from 13 to 8; ISI score decreased from 11 to 7.

ADT—antidepressant therapy; QIDS-SR16—Quick Inventory of Depressive Symptomatology Self-Report (16-item); MADRS—Montgomery–Åsberg Depression Rating Scale; PSQI—Pittsburgh Sleep Quality Index; ISI—Insomnia Severity Index; MDE—major depressive episode; BPD—bipolar disorder; BDNF—brain-derived neurotrophic factor, EEG—electroencephalography; IV—intravenous; TRD—treatment-resistant depression; SD—standard deviation; SEM—standard error of the mean.

**Table 2:** Non-randomised study evaluated using Newcastle–Ottawa Scale.

Study		Rodrigues 2022 [40]
Selection	Representativeness of the exposed cohort	1
	Selection of the non-exposed cohort	−
	Ascertainment of exposure	1
	Demonstration that outcome of interest was not present at start of study	1
Comparability	Comparability of cohorts on the basis of the design or analysis controlled for confounders	2
Outcome	Assessment of outcome	−
	Was follow-up long enough for outcomes to occur	1
	Adequacy of follow-up of cohorts	1
Total		**7**

## Data Availability

The search strategy is presented in the methodology section and on the flow chart.

## Supplementary Materials

The following supporting information can be downloaded at: https://www.mdpi.com/article/10.3390/ph16040568/s1, Prisma checklist, raw PubMed results.
